# Mechanistic Insights into Full Solid-Waste Activators for Enhancing the Performance of Blast Furnace Slag–Fly Ash Cementitious Composites

**DOI:** 10.3390/ma18143275

**Published:** 2025-07-11

**Authors:** Huiying Zhang, Yongchun Li, Dingbang Wei, Xu Wu, Yapeng Wang

**Affiliations:** 1 Gansu Provincial Highway Development Group Co., Ltd., Lanzhou 730030, China; 18189547190@163.com; 2 Gansu Provincial Transportation Planning Survey & Design Institute Co., Ltd., Lanzhou 730030, China; 13639336457@163.com (D.W.); 18893105922@163.com (X.W.); 19994163665@163.com (Y.W.); 3 School of Civil Engineering, Lanzhou Jiaotong University, Lanzhou 730070, China

**Keywords:** alkali-activated materials, solid waste recycling, slag–fly ash composites, hydration mechanisms, chloride immobilization

## Abstract

To address the practical limitations of conventional alkaline activators (e.g., handling hazards, cost) and promote the resource utilization of industrial solid wastes, this study developed a novel all-solid-waste activator system comprising soda residue (SR) and carbide slag (CS). The synergistic effects of SR-CS activators on the hydration behavior of blast furnace slag (GGBS)–fly ash (FA) cementitious composites were systematically investigated. Mechanical performance, phase evolution, and microstructural development were analyzed through compressive strength tests, XRD, FTIR, TG-DTG, and SEM-EDS. Results demonstrate that in the SR-CS activator system, which combines with desulfuriation gypsum as sulfate activator, increasing CS content elevates the normal consistency water demand due to the high-polarity, low-solubility Ca(OH)_2_ in CS. The SR-CS activator accelerates the early hydration process of cementitious materials, shortening the paste setting time while achieving compressive strengths of 17 MPa at 7 days and 32.4 MPa at 28 days, respectively. Higher fly ash content reduced strength owing to increased unreacted particles and prolonged setting. Conversely, desulfurization gypsum exhibited a sulfate activation effect, with compressive strength peaking at 34.2 MPa with 4 wt% gypsum. Chloride immobilization by C-S-H gel was confirmed, effectively mitigating environmental risks associated with SR. This work establishes a sustainable pathway for developing low-carbon cementitious materials using multi-source solid wastes.

## 1. Introduction

The rapid industrialization in China from the mid-20th century to the present has generated substantial industrial solid wastes, primarily comprising ground granulated blast furnace slag (GGBS) from steel plants and fly ash (FA) from thermal power plants. Recent statistics reveal an annual production of approximately 3.7 billion tons of industrial solid waste, with a comprehensive utilization rate of merely 55% and accumulated stockpiles exceeding 60 billion tons [1]. Conventional disposal methods, such as landfilling and roadbed backfilling, not only result in resource wastage and economic burdens for enterprises but also pose significant environmental risks. Meanwhile, as the necessity for human society, cement production accounts for 7–8% of global CO_2_ emissions from human behaviors [2]. This has caused problems like global warming and glacier melting. Fortunately, most industrial solid wastes, predominantly composed of Si, O, and Al elements, exhibit potential hydration activity or geopolymerization capabilities under aqueous conditions. These materials can serve as cementitious alternatives to traditional Portland cement without requiring energy-intensive “two-step grinding and one-step calcination” processes [3], presenting crucial implications for achieving carbon neutrality and sustainable development.

Extensive research has been conducted on alkali-activated binders (AABs) in recent years. Korniejenko, K. et al. [4] reviewed the global literature on geopolymer production from industrial solid wastes for circular economy objectives, revealing that the role of industrial solid wastes in the construction materials sector is increasing, as they constitute valuable sources for geopolymer synthesis. The development of sustainable materials enables post-service life reuse, thereby introducing closed-loop processes into production systems—a critical pathway toward achieving circular economies. Xie et al. [5] systematically investigated red mud–fly ash binary geopolymer materials using sodium silicate and NaOH as activators, revealing optimal workability, stability, and compressive strength at an activator modulus of 3.0. Liu et al. [6] developed a ternary geopolymer system (FA–slag–carbide slag = 32:15:3), achieving 77.83 MPa compressive strength at 28 days through optimized parameters (solid–liquid ratio 0.55, activator modulus 1.2), with microstructural characterization via MIP and SEM. Cai et al. [7] compared the durability of ultra-high-strength alkali-activated concrete (AAB-UHSC) with conventional counterparts, demonstrating superior chloride resistance but relatively weaker carbonation resistance compared to OPC-UHSC, while noting sixfold enhanced carbonation resistance versus AAB-NSC. Xia et al. [8] formulated high-performance AABs (>70 MPa) using desert sand and high-calcium FA, identifying optimal parameters (alkali content 5–8%, sodium silicate modulus 1.0–1.5) and attributing strength development to C(N)-A-S-H gel formation.

**Figure 1:**
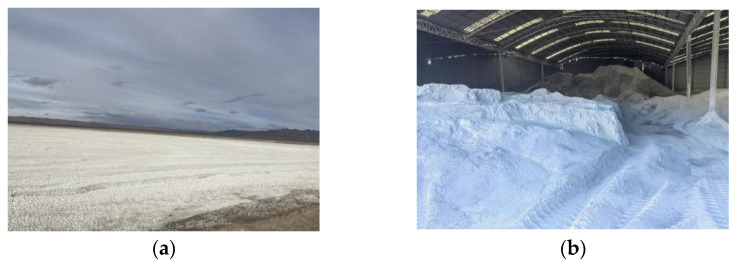
Large piles of alkaline solid waste: (**a**) SR, (**b**) CS.

Current research predominantly employs strong alkaline solutions (NaOH/sodium silicate) as activators, which present limitations for road engineering applications requiring “dry-mix transportation and on-site mixing.” Moreover, these chemical activators incur high costs. Recent investigations have explored alkaline solid wastes as alternative activators, including carbide slag [9,10,11,12] and red mud [13,14]. In Qinghai Province, substantial quantities of soda residue (SR, pH > 13) from sodium carbonate production (Figure 1a) and carbide slag (CS, Ca(OH)_2_-dominated, pH > 13, Figure 1b) from acetylene manufacturing accumulate, creating significant environmental and economic pressures.

This study innovatively develops a multi-component solid-waste-based activator system using GGBS and FA as precursors, supplemented with SR (alkaline activator) and desulfurization gypsum (sulfate activator). Compared to traditional alkali activators, this study eliminates chemical reagents by utilizing industrial solid wastes for both precursor materials and activators in the cementitious system. This novel approach offers simplified production processes, cost-effectiveness, and a low carbon footprint. In the study, we systematically investigate the synergistic effects of various activator combinations on the mechanical properties of the GGBS-FA binary system. Advanced characterization techniques, including XRD, SEM-EDS, FTIR, and TG-DSC, are employed to elucidate the activation mechanisms and hydration products formation.

## 2. Materials and Methods

### 2.1. Raw Materials

The chemical compositions of raw materials are presented in Table 1. The desulfurization gypsum (gypsum) and ground granulated blast furnace slag (GGBS) were supplied by Lanxin Iron & Steel Co., Ltd. (Lanzhou, China), while fly ash (FA) was obtained from Lanzhou Hongyuan Building Materials Co., Ltd. (Lanzhou, China). Carbide slag (CS) and soda residue (SR) were provided by Qinghai Salt Lake Magnesium Industry Co., Ltd. (Golmud, China). The SR was initially in a wet state with 65% moisture content, exhibiting a milky-white appearance. After natural drying, the pH value was measured at 11 using the ion-selective electrode method. All other materials (CS, gypsum, GGBS, and FA) were supplied in dry form. The GGBS demonstrates a density of 2.9 g/cm^3^ and a specific surface area of 424 m^2^/g, with 17.5% residue on a 45 μm sieve for FA.

Based on the chemical composition of the GGBS in Table 1, the basicity coefficient (Mo) and quality coefficient (K) were calculated using Equations (1) and (2), yielding M_O_ = 0.86 (confirming the alkaline nature of the slag) and K = 1.87, which complies with GB/T 18046-2017 “Ground granulated blast furnace slag for cement, mortar, and concrete” [15]. Activity index validation per this standard demonstrated a 7-day activity index of 70.2% and a 28-day index of 96%, qualifying the material as S95-grade slag powder with high reactivity.

(1) $$M_{O}=\frac{\mathrm{CaO}+\mathrm{MgO}}{{\mathrm{SiO}}_{2}+{\mathrm{Al}}_{2}O_{3}}$$

(2) $$K=\frac{\mathrm{CaO}+\mathrm{MgO}+{\mathrm{Al}}_{2}O_{3}}{{\mathrm{TiO}}_{2}+{\mathrm{SiO}}_{2}+\mathrm{MnO}}$$

**Figure 2:**
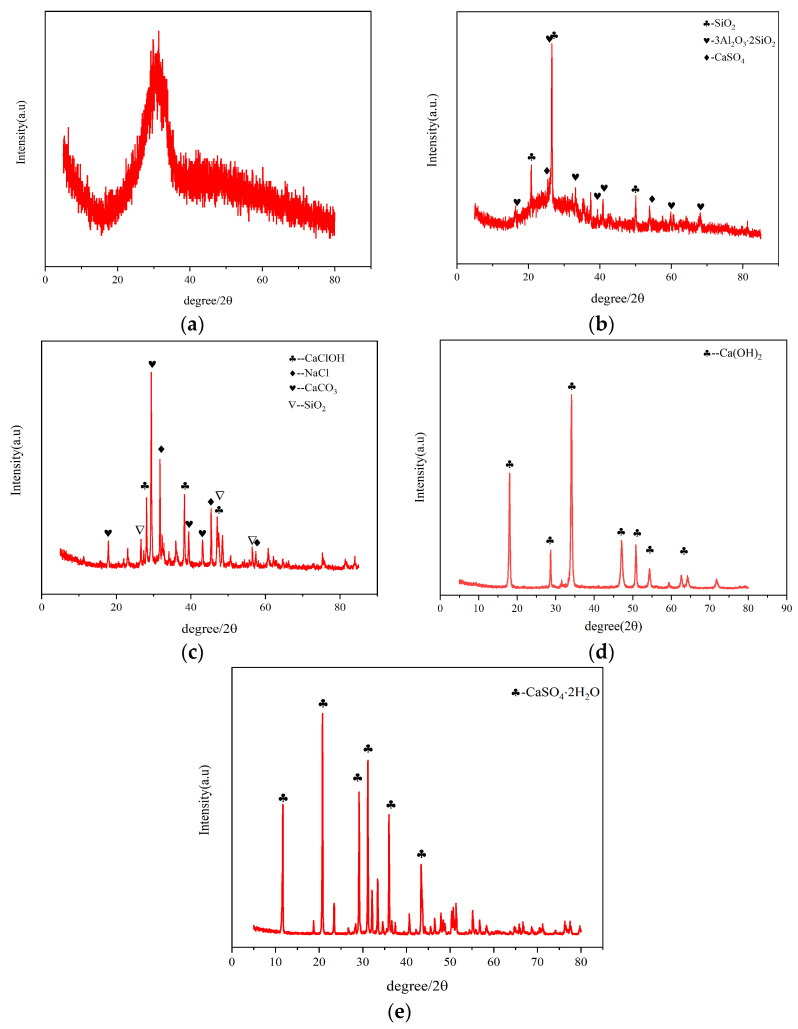
XRD: (**a**) GGBS, (**b**) FA, (**c**) SR, (**d**) CS, (**e**) gypsum.

X-ray diffraction (XRD) analysis (Figure 2) revealed distinct phase characteristics:

- GGBS (Figure 2a) displayed a broad hump between 20 and 40° 2θ, indicating predominant amorphous glass phases with high reactivity.
- FA (Figure 2b) contained crystalline quartz (SiO_2_) and mullite (3Al_2_O_3_·2SiO_2_) with secondary phases of anhydrite (CaSO_4_), accompanied by reactive glassy phases.
- SR (Figure 2c) primarily consisted of Calcite (CaCO_3_), Common salt (NaCl), and Calcium chloride (CaClOH) phases formed through complex precipitation processes involving Ca^2+^, Cl^−^, CO_3_^2−^, and Na^+^ ions.
- CS showed a dominant Ca(OH)_2_ phase (91.66% CaO content).
- Gypsum was identified as dihydrate calcium sulfate (CaSO_4_·2H_2_O).

**Figure 3:**
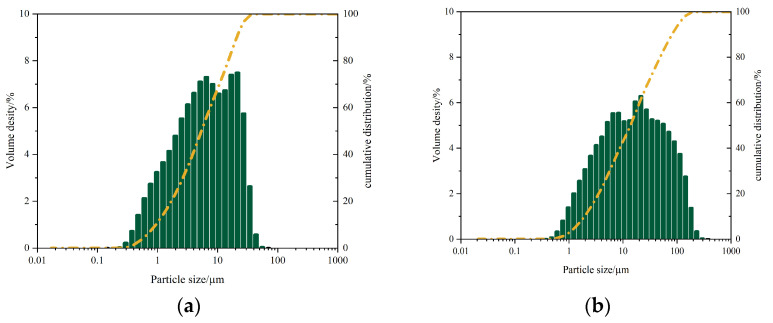
Particle size distribution: (**a**) GGBS, (**b**) FA.

Particle size distribution analysis using a Shimadzu SALD-2300 laser particle size analyzer (Shimadzu, Shanghai, China) (Figure 3) demonstrated the following:

- GGBS: median diameter (D_50_) = 5.40 μm, mean diameter = 4.91 μm, mode diameter = 19.02 μm, SD = 0.49;
- FA: D_50_ = 13.74 μm, mean diameter = 12.89 μm, mode diameter = 19.02 μm, SD = 0.59.

### 2.2. Mix Proportion Design

Previous studies have established optimal dosage ranges of 4–13 wt% for carbide slag (CS) and 15–30 wt% for soda residue (SR) as activators. This investigation develops a compound activator system (SR:CS = 3:7 by mass ratio) through a three-phase experimental design:

(1). Phase I—Activator optimization:
  - Baseline groups (BG-series) with GGBS as sole precursor;
  - CS: 4/8/12 wt%;
  - SR: 26/22/18 wt%;
  - Constant GGBS content: 70 wt%.
(2). Phase II—Binary precursor system:
  - Optimal activator combination with FA substitution (10/20/30 wt%, OG-series).
(3). Phase III—Sulfate activation:
  - Gypsum substitution (4–10 wt%, SOG-series) in FA-containing system

### 2.3. Specimen Preparation

(1). Precisely weigh constituents according to Table 2 proportions.
(2). Sequentially add materials to the pre-wetted mortar mixer:
  - Initial low-speed mixing (140 ± 5 rpm): 30 s binder–water blending;
  - Standard sand incorporation during the second 30 s of low-speed mixing;
  - High-speed mixing (285 ± 10 rpm): 30 s + 60 s after 90 s rest period.
(3). Cast 40 × 40 × 160 mm prism specimens using two-layer placement.
(4). Compact each layer with 60 vibrations on the standard jolting table.
(5). Cure under controlled conditions (20 ± 2 °C, 95% RH) for 24 h prior to demolding.

### 2.4. Testing Protocols

- Strength development: Measure 3/7/28-day compressive strength per GB/T 17671 (ISO 679) [16];
- Phase analysis: Rigaku D/max-A XRD (Tokyo, Japan) (Cu-Kα, 40 kV/40 mA, 5–85° 2θ, 2°/min);
- Thermal analysis: Shimadzu DTG-60 AH (Shanghai, China) (N_2_, 50 mL/min, 10 °C/min to 900 °C);
- Molecular characterization: Nicolet iS5 FTIR (Beijing, China) (400–4000 cm^−1^, KBr pellet);
- Microstructural observation: ZEISS Sigma300 SEM (Jena, Germany) (90 s Au-sputtered samples).

### 2.5. Statistical Analysis

Statistical analysis was performed using IBM SPSS Statistics (Version 31). The compressive strength and bending strength results obtained at 7 days and 28 days of curing are presented as mean ± standard deviation (SD), with each data point representing the mean of measurements from *n* = 11 independent specimens per age. The normality of the data distribution within each age group was assessed using the Shapiro–Wilk test (α = 0.05), and the homogeneity of variances between the two age groups was evaluated using Levene’s test (α = 0.05). An independent samples *t*-test was used to determine if there was a statistically significant difference in mean strength between the 7-day and 28-day curing periods; a probability (*p*) value of less than 0.05 (*p* < 0.05) was considered statistically significant. The error bars depicted in the bar charts represent the standard deviation (SD).

## 3. Results and Discussion

### 3.1. The Influence of Multi-Source Solid-Waste Activation on the Standard Consistency Water Demand and Setting Time of the GGBS-FA System

**Figure 4:**
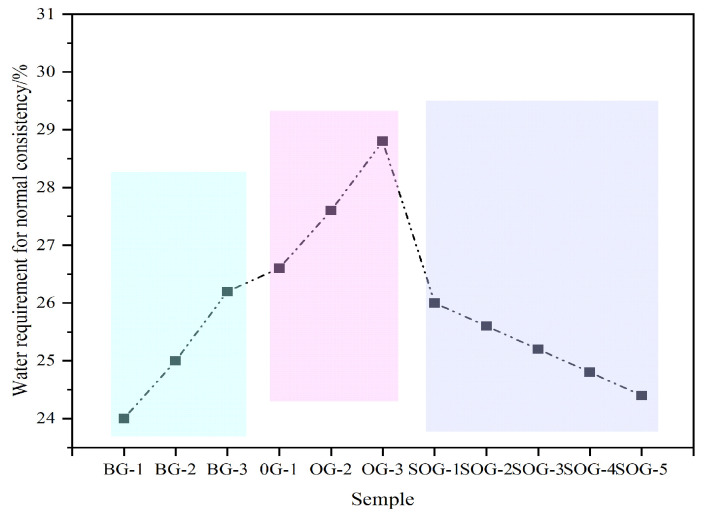
Influence of multi-source solid-waste activation on the standard consistency water demand of the GGBS-FA system.

Figure 4 illustrates the influence of multi-source solid-waste activation on the standard consistency water demand of cementitious material systems. In the BG system, as the CS content increases, the standard consistency water demand of the paste rises from 24% to 26.2%. This occurs because CS primarily consists of highly polar and low-solubility Ca(OH)_2_, whose loose and porous microstructure results in a large specific surface area. Compared to SR, CS requires more water to wet particle surfaces and fill pores. In the OG system, with increasing FA substitution for GGBS, the standard consistency water demand of the paste increases from 26.2% to 28.8%. This is mainly attributed to FA’s porous or hollow particle morphology and higher specific surface area, which demand additional water for surface wetting and pore filling. In contrast, GGBS effectively combines free water and optimizes paste fluidity through its dense particle structure. In the SOG system, as gypsum replaces GGBS, the standard consistency water demand decreases from 28.8% to 24.4%. This reduction stems from gypsum’s composition, dominated by soluble calcium sulfate, whose dense particle morphology and ion dissolution release effectively reduce paste viscosity while diminishing free water requirements.

**Figure 5:**
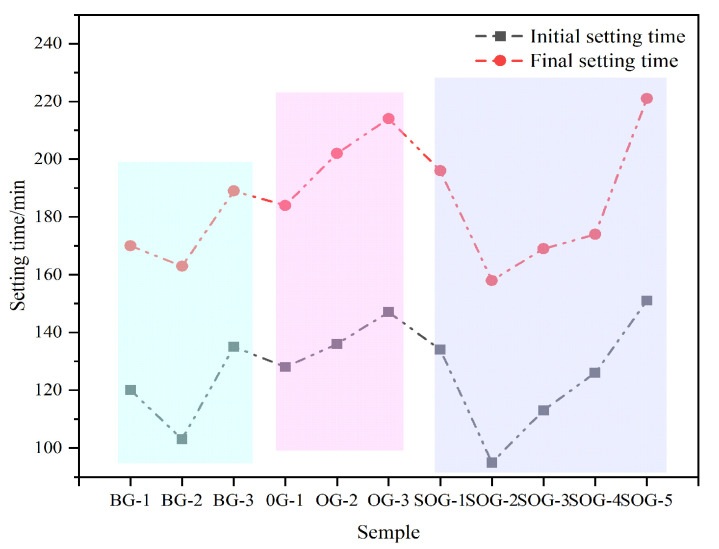
Influence of multi-source solid-waste activation on the setting time of the GGBS-FA system.

Figure 5 illustrates the influence of multi-source solid-waste activation on the setting time of cementitious material systems. In the BG system, the initial setting time of the paste first decreases and then increases, with BG-2 exhibiting the shortest initial setting time of 103 min. This is attributed to the optimal activation ratio achieved between CS and SR in the paste, which promotes hydration. In the OG system, as the FA substitution for GGBS increases, the setting time gradually lengthens, with OG-3 showing the longest initial setting time of 147 min. This occurs because the hydration activity of FA is significantly lower than that of GGBS, slowing the hydration process and prolonging the setting time. In the SOG system, the setting time first decreases and then increases with the substitution of gypsum for GGBS. At low substitution levels, gypsum provides a “sulfate activation” effect, accelerating hydration and shortening the setting time. However, as the substitution amount increases, excessive gypsum fails to participate in hydration reactions during the early stages, leading to an extended setting time.

### 3.2. Effects of Multi-Source Solid-Waste Activation on Mechanical Properties of Slag–Fly Ash System

**Figure 6:**
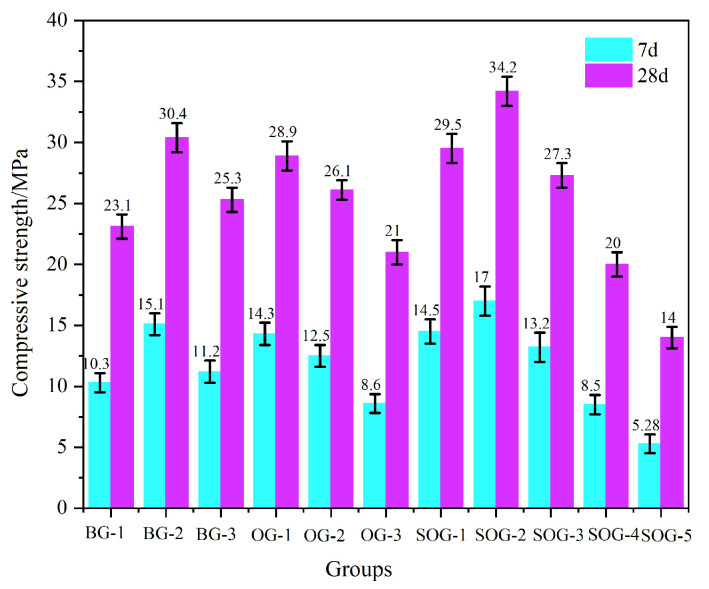
Compressive strength.

**Figure 7:**
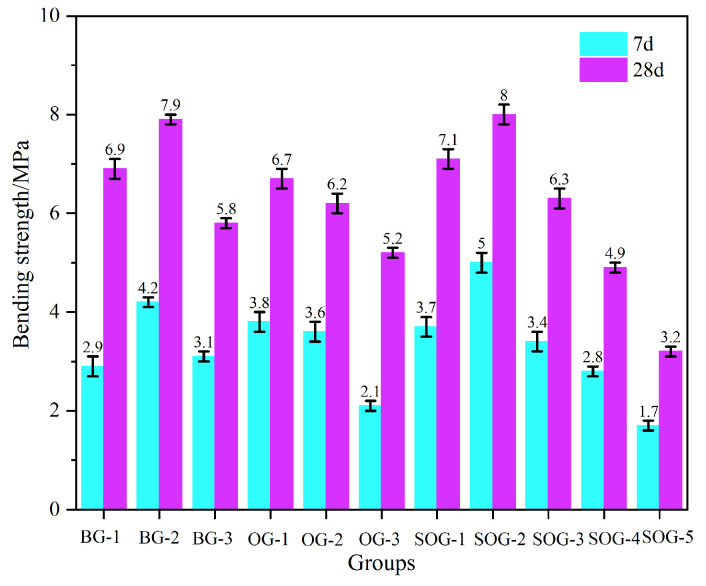
Bending strength.

Figure 6 and Figure 7 present the 7-day and 28-day compressive and flexural strengths of cement mortar under different mix proportions. The strength test results demonstrate that when the total content of alkali residue and carbide slag reaches 30 wt%, their activation effect on slag becomes feasible. As carbide slag replaces alkali residue in 4% wt increments, the mortar strength initially increases, then decreases. The optimal alkali residue content is 22 wt% with 8 wt% carbide slag, achieving a 28-day compressive strength of 30.4 MPa. This occurs because the alkaline environment created by alkali residue and carbide slag facilitates the “depolymerization” reaction of slag, promoting the formation of C-S-H and C-A-S-H gels during hydration, thereby enhancing strength development [17]. Increased carbide slag content introduces substantial Ca^2+^ and OH^−^ ions, establishing a strong alkaline environment and supplementing calcium ions to accelerate nucleation of C-S-H and C-A-S-H gels. Simultaneously, the supplemented Ca^2+^ promotes the formation of ettringite (AFt) from aluminosilicate phases in slag [18]. However, when carbide slag content increases from 8 wt% to 12 wt%, the 28-day mortar strength decreases from 30.4 MPa to 25.3 MPa due to Ca(OH)_2_ solution saturation and subsequent crystal formation that impedes strength development [19].

Under optimal alkali residue and carbide slag proportions, when fly ash replaces slag in 10 wt% increments, both 7-day and 28-day strengths progressively decrease. At 30 wt% fly ash content, 7-day compressive strength decreases by 43% and 28-day strength by 30.9%. This reduction occurs because fly ash exhibits significantly lower hydration activity than slag powder under ambient conditions, slowing the hydration rate of the system [20,21].

In five SOG system test groups using desulfurization gypsum to replace slag powder in 2 wt% increments, strength tests reveal initial strength enhancement followed by reduction with increasing gypsum content. The maximum strength occurs at 4 wt% desulfurization gypsum content, yielding 17 MPa 7-day compressive strength and 34.2 MPa 28-day strength. Appropriate gypsum addition supplements Ca^2+^ and SO_4_^2−^ ions in the alkaline environment created by alkali residue and carbide slag, promoting AFt formation during slag–fly ash hydration. The interlocking needle-shaped AFt crystals form a reinforcing framework. However, excessive gypsum content induces expansion stress from unreacted gypsum during early hydration, increasing mortar porosity and reducing strength [22].

### 3.3. XRD Analysis

To ensure the relevance and comprehensiveness of this study, samples for microscopic analysis were selected to encompass the influence of the activator on three distinct cementitious material systems: GGBS alone, GGBS-FA, and GGBS-FA–gypsum. Furthermore, within each of these three systems, the specimen exhibiting optimal mechanical performance at both the 7-day and 28-day curing ages (specifically BG-2, OG-1, and SOG-2, respectively) was chosen for microscopic analysis.

**Figure 8:**
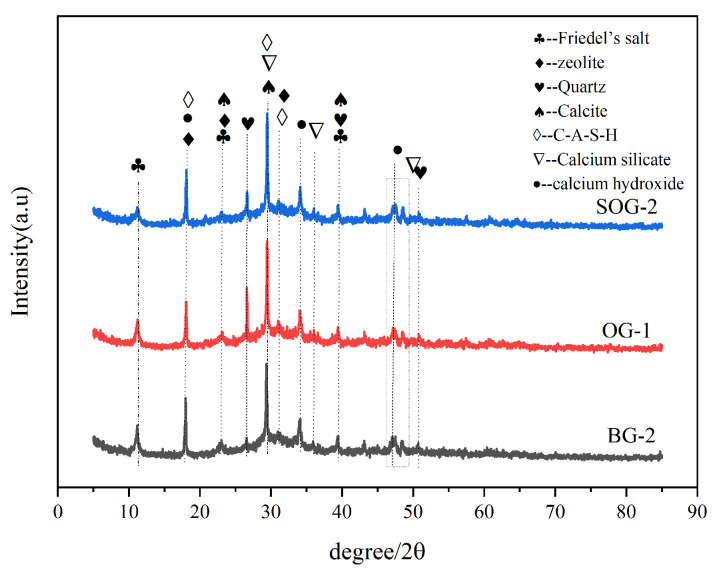
XRD patterns of 28-day specimens.

To characterize the types of hydration products formed with different precursors and activators, XRD analysis was conducted on three groups of samples (BG-2, OG-1, and SOG-2) after 28 days of hydration, with results shown in Figure 8. The main crystalline phases identified in the three groups of samples after 28 days of hydration include Friedel’s salt (3CaO·Al_2_O_3_·CaCl_2_·10H_2_O, FS), calcite (CaCO_3_), quartz (SiO_2_), calcium aluminosilicate hydrate (C-A-S-H), calcium hydroxide, calcium silicate, and zeolite (Ca_2_(Si_9_Al_3_)O_24_·8H_2_O). Among these, FS, C-A-S-H, and zeolite are primarily generated through hydration reactions. The XRD patterns exhibit a distinct “hump” region between 20 and 40°, attributed to the formation of amorphous C-S-H gel during hydration [23]. A diffraction peak of C-A-S-H is observed near 31°, where Al^3+^ ions dissolved from Al_2_O_3_ in the precursor are incorporated into the C-S-H gel during hydration to form C-A-S-H [24]. Simultaneously, the Si-O and Al-O tetrahedra in slag powder and fly ash undergo “depolymerization-polycondensation” reactions in the alkaline environment created by alkali residue and carbide slag dissolution, producing zeolite-type minerals (Ca_2_(Si_9_Al_3_)O_24_·8H_2_O) [25].

Calcite mainly originates from alkali residue, quartz from unreacted slag powder and fly ash, and calcium hydroxide from carbide slag. The diffraction peak intensity of the quartz phase in OG-1 is significantly higher than that in BG-2 and SOG-2, with BG-2 showing the lowest intensity. This is attributed to the replacement of 10 wt% slag powder with fly ash in OG-1. Given the substantially lower cementitious activity of fly ash compared to slag powder, the unreacted quartz content in OG-1 exceeds that in BG-2 under equivalent alkali residue and carbide slag conditions. In SOG-2, the addition of 4 wt% desulfurization gypsum introduces Ca^2+^ and SO_4_^2−^ ions, creating a “sulfate activation” effect that enhances precursor hydration. Consequently, SOG-2 exhibits reduced quartz phase diffraction peaks compared to OG-1 [21].

### 3.4. FTIR Analysis

**Figure 9:**
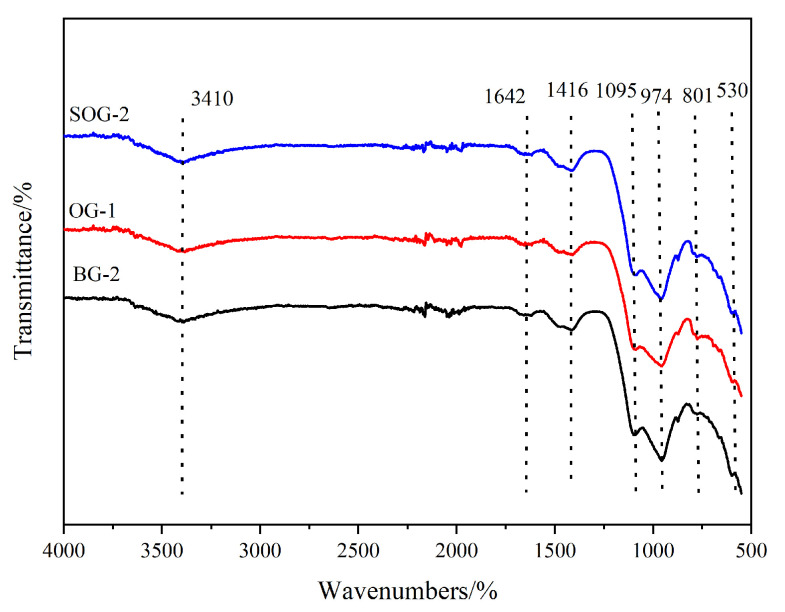
FTIR patterns of 28-day specimens.

The FTIR analysis results of 28-day hydration products for BG-2, OG-1, and SOG-2 are shown in Figure 9. Absorption bands near 530 cm^−1^, 801 cm^−1^, and 974 cm^−1^ correspond to stretching and bending vibrations of Si-O bonds, characteristic of C-S-H gel. SOG-2 exhibits the broadest and most intense absorption peaks at these positions, followed by BG-2, with OG-1 showing the weakest signals. This indicates that SOG-2 generates the highest amount of C-S-H gel after 28 days of hydration, consistent with its superior 28-day compressive strength results [26].

The absorption band near 1416 cm^−1^ arises from C-O bond stretching vibrations, confirming the presence of calcite [27]. Absorption bands at 1642 cm^−1^ and 3410 cm^−1^ are attributed to asymmetric stretching vibrations of O-H bonds, reflecting internal vibrations of crystalline water primarily originating from C-S-H gel and Friedel’s salt (FS) [26]. SOG-2 demonstrates the strongest intensity and broadest peaks for these features, followed by BG-2, while OG-1 shows the weakest signals. These observations suggest that SOG-2 contains the highest proportion of hydration products with crystalline water, aligning with its enhanced 28-day compressive performance compared to the other two groups.

### 3.5. TG-DTG Analysis

**Figure 10:**
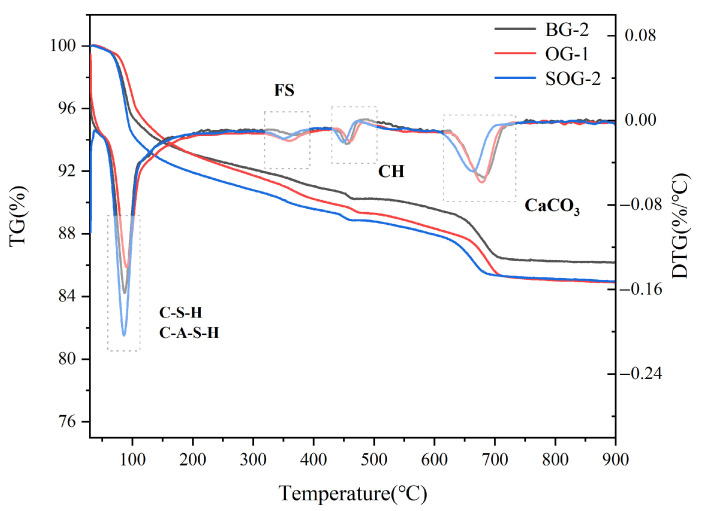
TG-DTG patterns of 28-day specimens.

TG-DTG analysis was conducted on 28-day hydrated samples of BG-2, OG-1, and SOG-2, with results shown in Figure 10. The DTG curves exhibit four primary weight loss peaks corresponding to the decomposition and dehydration of hydration products.

The weight loss peak in the 50–200 °C range primarily arises from the decomposition of C-S-H and C-A-S-H gels [28]. Within this temperature range, these gels undergo thermal decomposition and dehydration, leading to significant weight loss in the TG curves and corresponding peaks in the DTG curves. As shown in Figure 10, the DTG peak area in the 50–200 °C range follows the order SOG-2 > BG-2 > OG-1, indicating that SOG-2 produces the highest quantity of C-S-H and C-A-S-H gels during hydration, followed by BG-2 and then OG-1. This observation aligns with the 28-day compressive strength test results of the three groups.

The weight loss peak in the 300–400 °C range is attributed to the dehydration of Friedel’s salt (FS) [29]. Thermal dehydration of FS within this temperature range generates distinct DTG peaks, with peak areas following SOG-2 > BG-2 > OG-1, suggesting that FS formation during 28-day hydration follows the same order. These findings are consistent with XRD and FTIR results. The weight loss peak in the 450–550 °C range corresponds to the decomposition of Ca(OH)_2_ [10], primarily originating from unreacted calcium hydroxide in carbide slag, indicating incomplete consumption of Ca(OH)_2_ during hydration. The peak in the 600–750 °C range results from calcite decomposition [21], predominantly derived from alkali residue in raw materials, with a minor portion formed through carbonation of Ca(OH)_2_ during late hydration. Comparative analysis reveals that SOG-2 and BG-2 exhibit higher hydration degrees, resulting in less carbonation-induced calcite formation in SOG-2 [30].

In summary, the TG-DTG results demonstrate full consistency with XRD and FTIR experimental findings.

### 3.6. SEM-EDS Analysis

**Figure 11:**
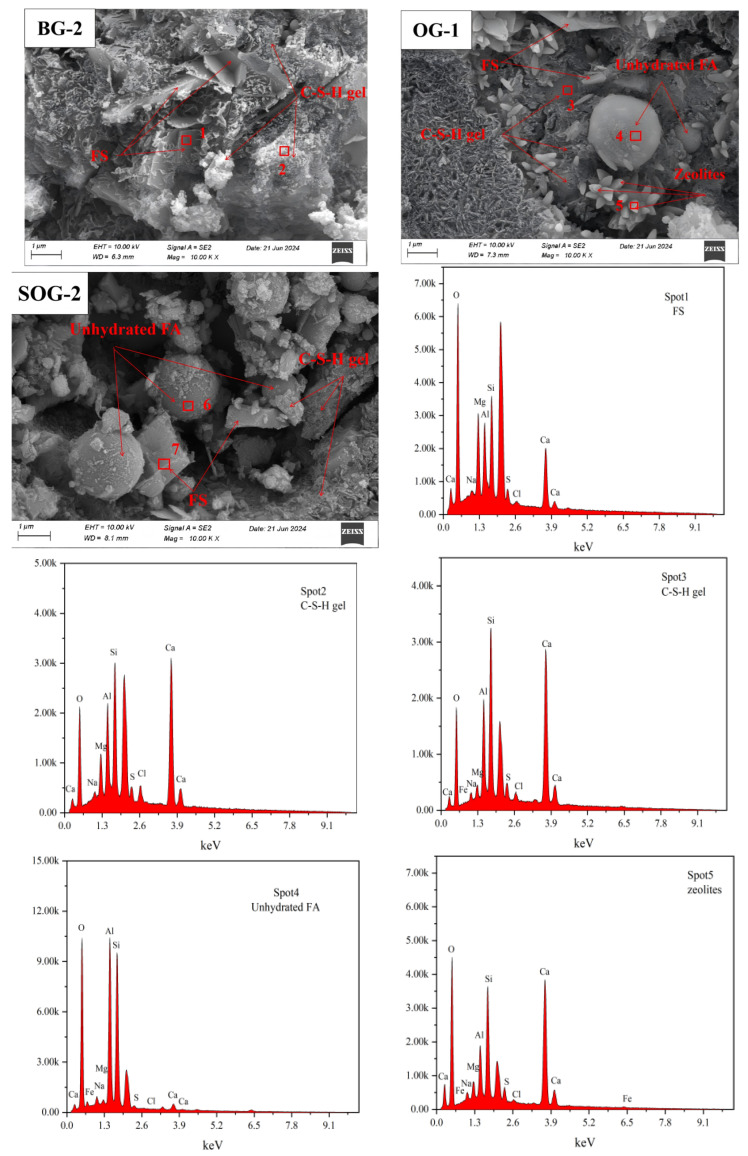

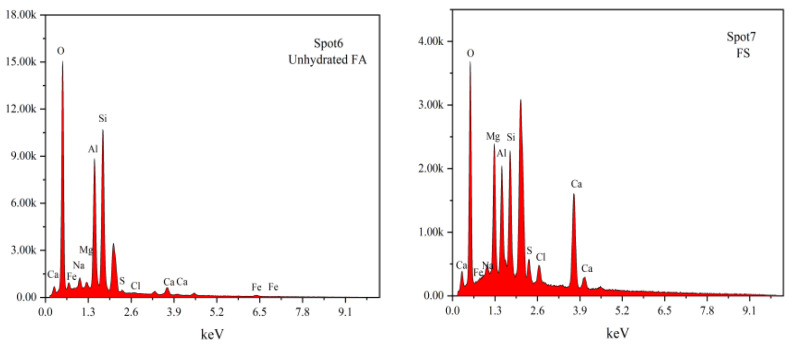
SEM-EDS test results of 28-day specimens.

To further clarify the hydration products of cementitious materials under different mixing ratios and analyze the influence of these ratios on their microstructure, SEM-EDS analysis was conducted on 28-day hydration products of three groups of samples (BG-2, OG-1, and SOG-2), as shown in Figure 11. The SEM-EDS results indicate that the primary hydration products of all three groups at 28 days are C-S-H gel. Among them, BG-2 exhibits the highest content of C-S-H gel, with a dense and uniformly distributed structure. Additionally, lamellar FS crystals are observed in BG-2, exhibiting a nested distribution within the C-S-H gel. This nested configuration constitutes a critical component of the cementitious strength [31], demonstrating that the optimal dosages of alkali residue and calcium carbide residue play a pivotal role in promoting the hydration of slag powder.

Compared to BG-2, the C-S-H gel structure in OG-1 appears looser, with significantly reduced gel content, thereby impeding strength development. SEM images reveal the presence of spherical and columnar phases. EDS analysis identifies the spherical particles as unhydrated fly ash, while the columnar phase corresponds to stilbite [29], consistent with XRD results.

In SOG-2, the hydration products primarily consist of C-S-H gel, FS crystals, and unhydrated fly ash particles. SEM images clearly show a denser C-S-H structure and increased FS content, attributed to the “sulfate activation” effect induced by the addition of desulfurization gypsum, which enhances sample strength. EDS results from Spot2 and Spot3 detect the presence of Mg, Al, and Cl elements, indicating partial substitution of Si^4+^ by Al^3+^ and Mg^2+^ in the C-S-H gel, along with Cl^−^ immobilization. Similar substitution patterns (partial replacement of Al^3+^ by Mg^2+^ and Si^4+^ in FS) are also observed in Spot1 and Spot7 [32].

In this study, we explore the possibility of all-solid-waste alkaline activator and systematically analyze the hydration products and microstructures of the cementitious composites. However, more work is needed to replenish. For example, TEM technique can be used to further investigate the generation of hydration products in the cementitious composite prepared in our study [33]; NMR can be quote to observe the molecular structural development of C-S-H [34,35]; MIP test can be invited to figure out the pore development in our cementitious composites [36,37]. Meanwhile, it is recognized that the high shrinkage associated with alkali-activated slag cementitious materials with high slag content is a major limitation for their practical application [38,39]. The incorporation of gypsum compensates for this slag hydration-induced shrinkage through the formation of AFt. However, excessive AFt formation or delayed AFt formation can lead to expansion and cracking. Therefore, further investigation into the gypsum content and ettringite formation in alkali-activated slag–gypsum cementitious systems is warranted [40,41,42,43].

## 4. Conclusions

This study investigated the effects of alkali residue–calcium carbide residue on the slag powder–fly ash precursor system through compressive strength analysis and microstructural characterization of hydration products. The following conclusions were drawn:

(1) When the alkali residue and calcium carbide residue contents reached 22 wt% and 8 wt%, respectively, the highest activation efficiency for slag powder was achieved. The compressive strength reached 15.1 MPa at 7 days and 30.4 MPa at 28 days. Substituting 10 wt% slag powder with fly ash resulted in reduced compressive strength at both curing ages (7 d and 28 d). This decline is attributed to the significantly lower hydration activity of fly ash under ambient conditions compared to slag powder, which decelerates the overall hydration kinetics of the system.
(2) Replacing slag powder with desulfurization gypsum at 2 wt% increments initially increased then decreased compressive strength. The maximum strength (17 MPa at 7 d and 34.2 MPa at 28 d) occurred at 4 wt% desulfurization gypsum content. This enhancement stems from the dissolution of gypsum, which releases Ca^2+^ and SO_4_^2−^ ions, inducing a “sulfate activation” effect that accelerates hydration reactions in the precursor system.
(3) XRD, FTIR, TG-DTG, and SEM-EDS analyses confirmed that the 28-day hydration products of BG-2, OG-1, and SOG-2 samples primarily consisted of FS, C-(A)-S-H gel, and zeolites. The quantities of FS and C-(A)-S-H gel followed the following order: SOG-2 > BG-2 > OG-1. Alkali residue and calcium carbide residue provided the necessary alkaline environment to promote slag hydration. Fly ash substitution (10 wt%) reduced hydration products due to its slower reaction kinetics, while subsequent 4 wt% desulfurization gypsum addition enhanced sulfate activation, increasing hydration product formation.
(4) The results demonstrate the feasibility of using a fully solid-waste composite activator (alkali residue–calcium carbide residue–desulfurization gypsum) to replace conventional strong alkali chemicals for activating slag powder–fly ash systems. This approach enables the development of low-carbon cementitious materials based entirely on solid wastes, offering significant potential for advancing green building materials.

## Figures and Tables

**Table 1:** Chemical composition of raw materials.

Material	CaO	SiO_2_	Al_2_O_3_	Fe_2_O_3_	MgO	TiO_2_	K_2_O	SO_3_	MnO	Na_2_O	Cl^−^	Other	LOI
GGBS	32.96	31.43	18.92	0.22	10.20	1.31	0.44	2.55	0.49	1.09	0.12	0.04	0.23
FA	9.58	44.82	28.24	5.39	1.68	0.98	1.68	1.74	0.09	3.30	0.21	0.38	1.93
SR	55.86	11.18	1.85	0.74	3.6	-	0.21	1.06	-	4.18	18.58	0.15	2.43
CS	91.62	5.12	1.99	0.27	-	0.05	-	0.76	-	-	-	0.06	0.11
Gypsum	34.04	3.64	1.98	0.60	1.19	0.07	0.26	39.37	0.02	0.25	0.13	0.08	18.53

**Table 2:** Mix ratio.

ID	SR (wt%)	CS (wt%)	GGBS (wt%)	FA (wt%)	Gypsum (wt%)	W/B
BG-1	26	4	70	-	-	0.5
BG-2	22	8	70	-	-	
BG-3	18	12	70	-	-	
OG-1	22	8	60	10		
OG-2	22	8	50	20		
OG-3	22	8	50	30		
SOG-1	22	8	58	10	2	
SOG-2	22	8	56	10	4	
SOG-3	22	8	54	10	6	
SOG-4	22	8	52	10	8	
SOG-5	22	8	50	10	10	

## Data Availability

The original contributions presented in this study are included in the article. Further inquiries can be directed to the corresponding author.
